# Hydrogen gas alleviates oxygen toxicity by reducing hydroxyl radical levels in PC12 cells

**DOI:** 10.1371/journal.pone.0173645

**Published:** 2017-03-31

**Authors:** Junchao Yu, Qiuhong Yu, Yaling Liu, Ruiyun Zhang, Lianbi Xue

**Affiliations:** 1 Department of Hyperbaric Oxygenation, Beijing Tiantan Hospital, Capital Medical University, Beijing, China; 2 Department of Neurology, Civil Aviation General Hospital, Beijing, China; University of PECS Medical School, HUNGARY

## Abstract

Hyperbaric oxygen (HBO) therapy through breathing oxygen at the pressure of above 1 atmosphere absolute (ATA) is useful for varieties of clinical conditions, especially hypoxic-ischemic diseases. Because of generation of reactive oxygen species (ROS), breathing oxygen gas at high pressures can cause oxygen toxicity in the central nervous system, leading to multiple neurological dysfunction, which limits the use of HBO therapy. Studies have shown that Hydrogen gas (H_2_) can diminish oxidative stress and effectively reduce active ROS associated with diseases. However, the effect of H_2_ on ROS generated from HBO therapy remains unclear. In this study, we investigated the effect of H_2_ on ROS during HBO therapy using PC12 cells. PC12 cells cultured in medium were exposed to oxygen gas or mixed oxygen gas and H_2_ at 1 ATA or 5 ATA. Cells viability and oxidation products and ROS were determined. The data showed that H_2_ promoted the cell viability and inhibited the damage in the cell and mitochondria membrane, reduced the levels of lipid peroxidation and DNA oxidation, and selectively decreased the levels of •OH but not disturbing the levels of O_2_^•-^, H_2_O_2_, or NO• in PC12 cells during HBO therapy. These results indicated that H_2_ effectively reduced •OH, protected cells against oxygen toxicity resulting from HBO therapy, and had no effect on other ROS. Our data supported that H_2_ could be potentially used as an antioxidant during HBO therapy.

## 1. Introduction

Oxygen gas has been present around the earth for 345 million years. It is essential for aerobic organisms to generate energy during respiration. However, anoxia plays an important role in the initiation and progression of various clinical conditions, leading to many hypoxic-ischemic diseases. Oxygen gas has been used in therapy for varieties of diseases. Hyperbaric oxygen (HBO) therapy, defined as the inhalation of 100% oxygen gas at a pressure greater than 1 atmosphere absolute (ATA), can increase oxygen tension in arterial blood and tissue, improve the cellular oxygen supply by raising the tissue-cellular diffusion gradient. It is also beneficial to treat air embolism, soft tissue infections, radiation necrosis, impaired wound healing, and decompression sickness.

HBO therapy, however, has several adverse consequences that limit its use in hospital. Breathing oxygen at high pressures for sufficient duration can cause oxygen-induced damages in central nervous system (CNS), ranging from mild neurological symptoms to severe tonic-clonic convulsion[1]. The oxygen-derived radicals may account for such damage[2]. Reactive oxygen species (ROS), including superoxide anion (O_2_^•-^**)**, hydrogen peroxides (H_2_O_2_), hydroxyl radical (•OH) which has the very strong oxidative capability, and nitric oxide (NO•), are generated and lead to toxicity during HBO therapy[3–5]. Antioxidants can prevent damage from the detrimental effect of ROS. However, at present, there is no effective antioxidant used in clinical practice. The use of oxygen gas in treating hypoxic-ischemic diseases is limited.

Recently, studies by Ohsama et al. (2007) revealed that molecular Hydrogen (diHydrogen, H_2_) could efficiently reduce •OH and attenuate oxidative stress and brain ischemic-reperfusion injury and it had no effect on other ROS such as O_2_^•-^, H_2_O_2_, and NO•[6]. This finding aroused the attention of scholars immediately after it was published, H_2_ was also confirmed as an antagonist to ROS from ischemia–reperfusion injury in the brain, spinal cord[7], myocardium[8], liver, intestine[9], retina, testis[10], and kidney[11]. Moreover, H_2_ also could be used to treat varieties of other diseases related to oxidative stress, such as traumatic[12], neurodegenerative disease[13], inflammatory disease[14], organ transplantation, metabolic syndrome[15], diabetes mellitus[16], sepsis[17], burn wounds[18], adverse reactions after chemotherapy[19], radiation-induced injury[20], hearing disorders, preeclampsia[21]. However, whether H_2_ can prevent the damage from the detrimental effect of ROS during HBO therapy and alleviate oxygen toxicity is not clear.

In this study, we investigated the effects of H_2_ on cell viability and integrity as well as the ROS during HBO therapy using PC12 cells. We found that H_2_ increased the cell viability and integrity of PC12 cells and decreased •OH levels during HBO therapy. Our finding provides a clue to potentially use H_2_ as an antioxidant during HBO therapy.

## 2. Materials and methods

### 2.1. Reagents

RPMI 1640, fetal bovine serum (FBS) and horse serum (HS), 0.25% Trypsin-EDTA solution were purchased from Hyclone (Logan, UT, USA). Poly-L-lysine (PLL), 4′,6-Diamidino-2-phenylindole dihydrochloride (DAPI), Paraformaldehyde were purchased from Sigma-Aldrich (St Louis, MO, USA). MitoSOX Red mitochondrial superoxide indicator, ROS Detection reagents, TMRM (Tetramethylrhodamine methyl ester), and MitoTracker Green (MTGreen) were purchased from Molecular Probes (Invitrogen, Carlsbad, CA). Hydroxyphenyl fluorescein (HPF) were purchased from Cell Technology Inc (Mountain View, CA, USA). 4,5-diaminofluorescein diacetate (DAF-2 DA) were purchased from Molecular Probes Inc(Eugene, OR, USA). Dimethyl sulfoxide (DMSO) were purchased from Solarbio Biotechnology (Beijing, China). 8-hydroxy-2'-deoxyguanosine (8-OH-dG) were purchased from R&D (Minneapolis, NE, USA). All other chemicals were of analytical grade.

### 2.2. Cell culture and treatment

The PC12 cell line was obtained from the Type Culture Collection of the Chinese Academy of Sciences, Shanghai, China. PC12 cells were cultured in RPMI 1640 medium supplemented with 10% HS, 5% FBS, and maintained in a humidified incubator containing 5% CO_2_ at 37°C. Culture medium was replaced with fresh medium every 2–3 days. After reaching 80% confluence, cells were collected and suspended at a density of 5x10^4^cells/ml in fresh medium, and seeded on plastic culture plates coated with PLL.

PC12 cells in logarithmic growth phase were randomly divided into 4 groups and placed in experimental pressure chambers, which were respectively supplemented with different gas: 1, Normobaric air group (Air group). PC12 cells were supplemented with air (consisting of 80% nitrogen, 20% oxygen, v/v%). 2, Normobaric hydrogen-air mixed gas group (Air-H_2_ group). PC12 cells were supplemented with hydrogen-air mixed gas (consisting of 78% nitrogen, 20% oxygen, and 2% Hydrogen, v/v%) at 1 ATA. 3, Hyperbaric oxygen group (HBO group). PC12 cells were supplemented with 100% oxygen at 5 ATA. 4, Hyperbaric oxygen-hydrogen mixed gas group (H_2_ group). The chamber was filled with hydrogen and oxygen mixed gas (the volume ratio of Hydrogen and oxygen is 2%:98%). Because of safety, the compression and decompression were conducted at a rate of 0.13ATA per min, slower than those in the previous studies[22]. Pressure in chamber was stabilized for 2h after it reached 5 ATA. The total time through the course was 3 h. Gas flow through the chamber was maintained at 3 L/min. Temperature in the chamber was maintained at 22–24°C, with relative humidity 60%.

### 2.3. Cell viability assay

PC12 cells was seeded in the 96-well plates (1x10^4^ cells per well) and cultured for 24 h. Then the cells were treated with different gases. After treatment, the viability of PC12 cells was measured using the WST-1 cell proliferation and cytotoxicity assay kit (Beyotime Biotechnology, Nanjing, China) according to the manufacturer’s instructions. The OD values were determined at the wavelength 450 nm and reference wavelength of 690 nm.

### 2.4. Determination of Lactate Dehydrogenase (LDH) leakage

LDH is released into culture medium when cell membrane is damaged, representing the injury of cells and the extent of loss of cell membrane integrity. The LDH activity in the medium was determined using a kit from JianCheng Bioengineering Institute (Nanjing, China). PC12 cells were seeded in 6-well plates coated with PLL and exposed to the experimental gases for 3 h. Cell-free culture medium were collected from each well and incubated with the appropriate reagents in the kit according to the supplier’s instructions. The intensity of red color formed in the assay was measured at a wavelength 440 nm.

### 2.5. Determination of the lipid peroxidation and nucleic acid oxidation levels

Maleic Dialdehyde (MDA) is generated from cell membrane lipid oxidation and generally considered as an indicator of lipid peroxidation. MDA levels were determined using a TBARS assay kit from JianCheng Bioengineering Institute (Nanjing, China). PC12 cells were seeded in 6-well plates, and exposed to the experimental gases. Cell-free culture medium was collected immediately for measuring the levels of MDA. The OD values were obtained at the wavelength 532 nm.

DNA can be damaged by ROS, generating many oxidative products including 8-OH-dG which is considered the popular biomarker for estimating the oxidative DNA damage. PC12 cells were seeded in 6-well plates, and exposed to the experimental gases. Cell-free culture medium was collected immediately for measuring the levels of 8-OH-dG. Content of 8-OH-dG in the culture was determined using a kit according to the manufacturer’s protocol. Plates were read at 450 nm. The concentrations of 8-OH-dG in the samples were determined using the standard curve for 8-OH-dG between 0 and 20 ng/ml.

### 2.6. Determination of mitochondrial membrane potential using fluorescence

The mitochondrial membrane potential (MMP) represents the integrity of the mitochondrial membrane. MMP was determined by using TMRM, which depends upon the mitochondrial membrane potential, and MTGreen, which are independent of the membrane potential. PC12 cells in logarithmic growth phase were exposed to the experimental gases for 3 h at 5 ATA. PC12 cells were then stained with TMRM and MTGreen for 20 min at 37°C after treatment. Cells were then washed twice with cold PBS and analyzed using a confocal laser scanning microscope (LAIKA, GERMANY). TMRM and MTGreen were respectively excited at 543nm and 490 nm, and the emissions were filtered using 580nm and 516 nm barrier filter.

### 2.7. Determination of ROS using fluorescent dyes

The •OH levels were determined using HPF which is a selective fluorescence dye for •OH. 10 uM HPF was added to PC12 cells in Glass-bottomed dishes coated with PLL and incubated for 30 min at 37°C. PC12 cells were exposed to different gases for 3 h. After washing with Hank's Buffered Salt Solution (HBSS), the cells were visualized using a confocal laser scanning microscope. HPF was excited at 488 nm and the emissions were filtered using a 515 nm barrier filter. The density of fluorescent signals was quantified from 100 cells of each experiment using US National Institutes of Health Image software.

The O_2_^•-^ levels in PC12 cells were measured using MitoSOX which is the mitochondrial superoxide indicator in HBSS containing calcium and magnesium, according to the manufacturer's recommendation. PC12 cells were loaded 5mM MitoSOX for 10 min in incubator at 37°C. Following dye loading, PC12 cells were washed twice with HBSS and supplemented with culture medium. Then the cells were exposed to different experimental gases for 3 h. MitoSOX-specific fluorescence was detected using a confocal laser scanning microscope with excitation at 510 nm and emission at 580 nm.

The H_2_O_2_ levels were determined using H_2_DCF-DA staining. PC12 cells were treated with H_2_DCF-DA dye in a final concentration of 10 mM for 20 min at 37°C according to the manufacturer’s protocol. The cells were washed three times with RPMI1640 and then placed in fresh medium. Then the cells were exposed to different gases for 3 h. DCF fluorescence was viewed and recorded using the confocal scanner with 488 nm excitation and 525 nm emission 3 h later.

The NO• levels were determined using DAF-FM DA fluorescence dye according to the manufacturer’s protocol. Briefly, PC12 cells were incubated with 5 uM DAF-FM DA at 37°C for 20 min, and washed three times with PBS to remove excess probe. the cells were exposed to the experimental gases for 3 h. The fluorescence intensity was measured by a confocal scanner with excitation at 495 nm and emission at 515 nm.

## 3. Statistical analysis

Statistical analysis was performed using SPSS 13.0 (SPSS, USA). Statistical significance of differences among the experimental groups were analyzed using one-way Analysis of Variance (ANOVA). *p*< 0.05 was considered significant.

## 4. Results

### 4.1. Hydrogen gas promoted the viability and inhibited the damage in the intergrity of PC12 cells during HBO therapy

**Fig 1:**
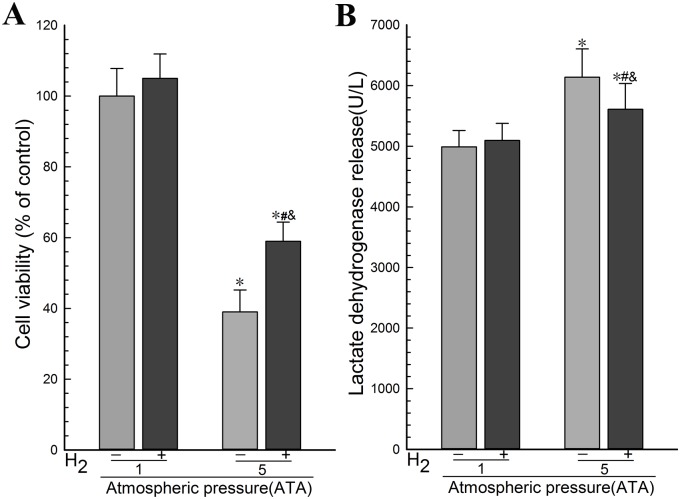
Hydrogen gas promoted the viability and inhibited the damage in the cell membrane of PC12 cells during HBO therapy. PC12 cells in logarithmic growth phase were exposed to the experimental gases for 3 h at 1 ATA or 5 ATA. (A) The viability of PC12 cells was determined using WST-1 assay. (B) The integrity of cell membrane in PC12 cells was determined using LDH assay. The data were analyzed by ANOVA followed by Fisher’s LSD test. *p<0.05 (n = 6), compared with the Air group, ^#^*p*<0.05 (n = 6^)^, compared with the HBO group, and ^&^*p*<0.05 (n = 6), compared with the Air-H_2_ group. Representative results were shown from at least three repeats.

To determine the effect of H_2_ on the viability and cell intergrity during HBO therapy, we exposed PC12 cells in logarithmic growth phase to the experimental gases and determined the viability of PC12 cells using WST-1 assay and the integrity of cell membrane in PC12 cells using LDH assay. The results showed that there was no significant difference in the cell viability between the Air group and the Air-H_2_ group (Fig 1A). Remarkable differences were found between the HBO group and the Air group (Fig 1A). The cellular viability in the HBO group was decreased more than that in the H_2_ group, and there was also a remarkable difference between H_2_ group and the Air group (Fig 1A). There was no significant differences in the concentration of LDH in the culture medium between the Air-H_2_ group and the Air group (Fig 1B). LDH leakage in the HBO group was increased remarkably, compared with the Air group. It was decreased in the H_2_ group, compared with the HBO group (Fig 1B). These results suggested that H_2_ promoted the viability and inhibited the damage in the cell membrane of PC12 cells during HBO therapy.

### 4.2. Hydrogen gas reduced the levels of lipid peroxidation and DNA oxidation in PC12 cells during HBO therapy

**Fig 2:**
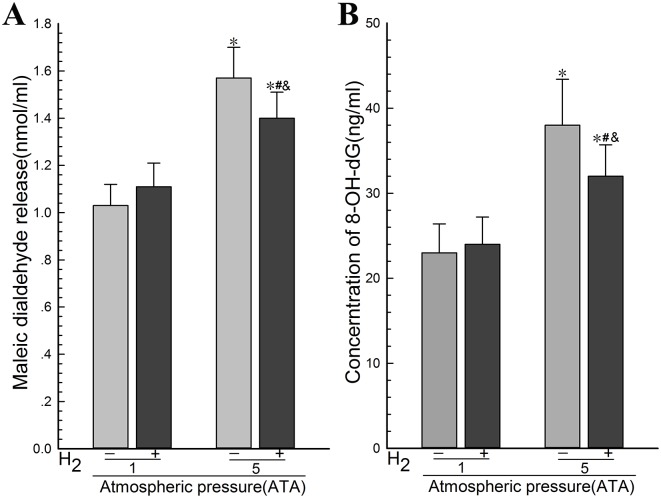
Hydrogen gas reduced the levels of lipid peroxidation and DNA oxidation in PC12 cells during HBO therapy. PC12 cells in logarithmic growth phase were exposed to the experimental gases for 3 h at 1 ATA or 5 ATA. (A) The MDA levels in the culture medium as an indicator for lipid peroxidation were determined using a TBARS assay kit. (B) The 8-OH-dG levels in the culture medium as an indicator for DNA oxidation were determined using a kit. The data were analyzed using ANOVA followed by Fisher’s LSD test. *p<0.05 (n = 6), compared with the Air group, ^#^*p*<0.05 (n = 6), compared with the HBO group, and ^&^*p*<0.05 (n = 6), compared with the Air-H_2_ group. Representative results were shown from at least three repeats.

To determine the effect of H_2_ on the lipid peroxidation and DNA oxidation in PC12 cells during HBO therapy, we exposed PC12 cells in logarithmic growth phase to the experimental gases and determined the MDA and the 8-OH-dG levels. The results showed that there was no significant difference in the MDA levels between the Air group and the Air-H_2_ group and remarkable differences were found between the HBO group and the Air group (Fig 2A). The MDA levels in the H_2_ group was lower than that in the HBO group which is higher than that of the Air group (Fig 2A). There was no significant difference in the content of 8-OH-dG in the culture medium between the Air-H_2_ group and the Air group (Fig 2B). The content of 8-OH-dG in the H_2_ group was lower than that in the HBO group (Fig 2B). These results suggested that H_2_ reduced the levels of lipid peroxidation and DNA oxidation in PC12 cells during HBO therapy.

### 4.3. Hydrogen gas protected mitochondria from HBO

**Fig 3:**
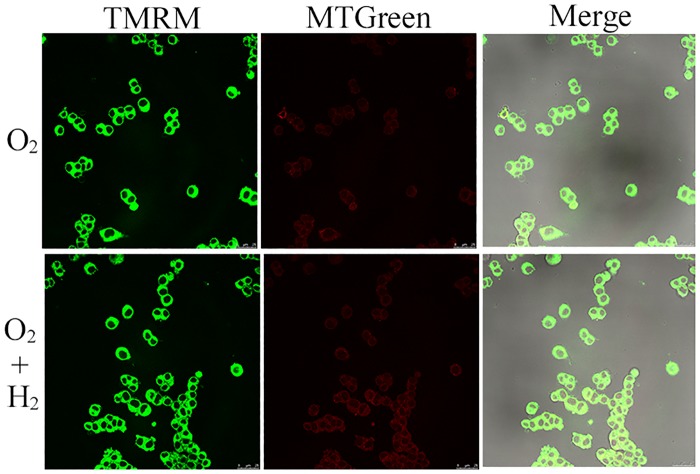
Hydrogen gas inhibited decrease in MMP levels of PC12 cells during HBO therapy. PC12 cells in logarithmic growth phase were exposed to the experimental gases for 3 h at 5 ATA and then stained using TMRM and MTGreen dyes. Fluorescence was detected using a Laser-Scanning Confocal Microscope. After HBO treatment, H_2_ prevented the decline of the mitochondrial membrane potential, as detected by fluorescence of TMRM, which depends upon the mitochondrial membrane potential, whereas fluorescence levels of MTGreen, which are independent of the membrane potential, were unchanged. Representative results were shown from at least three repeats.

To determine the effect of H_2_ on mitochondria in PC12 cells during HBO therapy, we exposed PC12 cells in logarithmic growth phase to the experimental gases and determined the levels of MMP using fluorescence. The results showed that the levels of MTGreen in the HBO group were significantly lower than those of the H_2_ group (Fig 3). But there was no significant difference in the levels of TMRM between the H_2_ group and the HBO group (Fig 3). These results suggested that H_2_ has a protective effect to mitochondria and inhibited decrease in MMP during HBO therapy.

### 4.4. Hydrogen gas selectively decreased the levels of •OH in PC12 cells during HBO therapy

**Fig 4:**
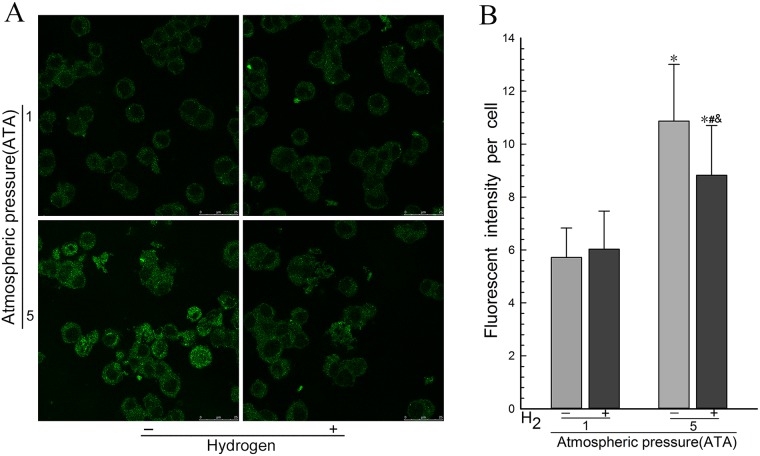
Hydrogen gas decreased the •OH levels in PC12 cells during HBO therapy. PC12 cells in logarithmic growth phase were exposed to the experimental gases for 3 h at 1 ATA or 5 ATA. The •OH levels (A and B) were determined using HPF staining. Fluorescence images of PC12 cells were obtained by Laser-Scanning Confocal Microscopy. Fluorescence was quantified from 100 cells of each independent experiment. *p<0.05, compared with the Air group. ^#^*p*<0.05, compared with the HBO group. ^&^*p*<0.05, compared with the Air-H_2_ group. Representative results were shown from at least three repeats.

**Fig 5:**
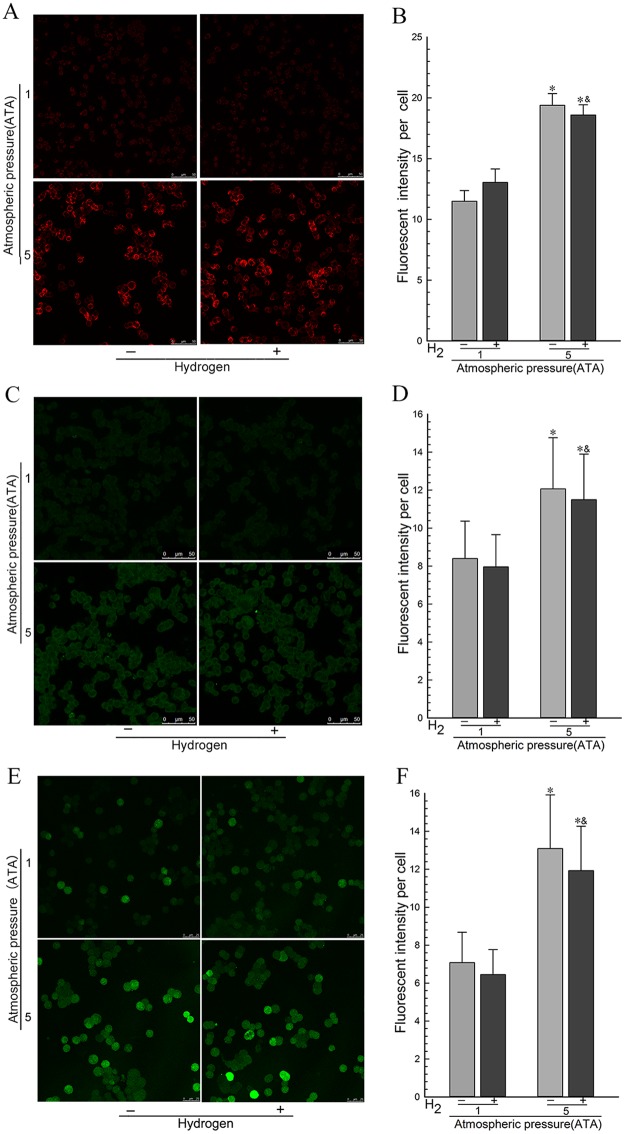
Hydrogen gas had no effect on the levels of O_2_^•-^, H_2_O_2_, and NO• in PC12 cells during HBO therapy. PC12 cells in logarithmic growth phase were exposed to the experimental gases for 3 h at 1 ATA or 5 ATA. The O_2_^•-^ levels (A and B), H_2_O_2_ levels (C and D), and NO• levels (E and F) were determined using MitoSOX, H_2_DCF-DA, and DAF-FM DA staining, respectively. Fluorescence images of PC12 cells were obtained by Laser-Scanning Confocal Microscopy. Fluorescence was quantified from 100 cells of each independent experiment. *p<0.05, compared with the Air group. ^&^*p*<0.05, compared with the Air-H_2_ group. Representative results were shown from at least three repeats.

To determine the effect of H_2_ on the ROS levels in PC12 cells during HBO therapy, we exposed PC12 cells in logarithmic growth phase to the experimental gases and determined the levels of •OH, O_2_^•-^, H_2_O_2_, and NO• using specific fluorescent dyes. The results showed that the levels of •OH in the HBO group were significantly higher than those of the Air group (Fig 4). It was significantly lower in the H_2_ group than those in the HBO group (Fig 4). The levels of O_2_^•-^, H_2_O_2_, and NO• in the HBO group or the H_2_ group were significantly higher than those of the Air group or the Air- H_2_ group (Fig 5), but there was no significant difference in the levels of O_2_^•-^, H_2_O_2_, and NO• between the H_2_ group and the HBO group or between the Air group and the Air-H_2_ group (Fig 5). These results suggested that H_2_ selectively decreased the levels of •OH but not disturbing the levels of O_2_^•-^, H_2_O_2_, and NO• in PC12 cells during HBO therapy.

## 5. Discussion

In the current study, we have found that H_2_ promoted the viability and inhibited the damage in the cell membrane and mitochondria, reduced the levels of lipid peroxidation and DNA oxidation, and H_2_ selectively decreased the levels of •OH but not disturbing the levels of O_2_^•-^, H_2_O_2_, and NO• in PC12 cells during HBO therapy. Our data support that H_2_ could be potentially used as an antioxidant during HBO therapy.

During hyperbaric oxygen therapy, excessive oxygen increases the production of O_2_^•-^ in living cells, especially in mitochondria, which is the most important site producing ROS. O_2_^•-^ is converted into H_2_O_2_ by superoxide dismutase, and H_2_O_2_ is converted into •OH via the Fenton or Weise reaction in the presence of catalytically active metals, such as Fe^2+^ and Cu^+^. •OH is one of the most reactive ROS and reacts indiscriminately with biological macromolecules, including lipids, proteins, and nucleic acids[23], inducing oxygen toxicity. During HBO therapy with 2% H_2_, Hydrogen is accompanied with oxygen all the time, and reaches terminal tissue after passing through grades of arteries[24]. Additionally, as the molecular Hydrogen is electrically neutral and much smaller than the oxygen molecule, it easily penetrates cell membranes and enters into cells and organelles, such as nucleus and mitochondria. When •OH is produced in cells, Hydrogen can react with it immediately to produce H_2_O before they attack biologically essential molecules. Therefore, Hydrogen can prevent the reaction between •OH and macromolecules, reducing the levels of oxidized macromolecules, decrease the magnitude of cell and mitochondria injury, prevent PC12 cells from oxygen toxicity resulting from hyperbaric oxygen. Hydrogen also inhibits oxidative stress by restoring the antioxidant capacity of superoxide dismutase, catalase and glutathione peroxidase, and alleviates the detrimental effects of hyperbaric oxygen.

In normal situations, the endogenous antioxidant network provides sufficient protection against ROS. It is necessary to supply exogenous antioxidants to counter abruptly increased ROS which overwhelms the capacity of endogenous antioxidants during hyperbaric oxygen therapy[25]. There are four types of insufficiency for antioxidants. Firstly, the physiological antioxidants scavenge multiple radicals, not only •OH, but also O_2_^•-^ and H_2_O_2_, which are involved in cell signaling and defense against harmful stimuli[26]. Second, some antioxidants such as Vitamin A, Vitamin C, and Vitamin E are efficacious in vitro experiments and animals, but not working in clinical practice[27]. Third, excess antioxidants may increase mortality and incidence of cancer[28,29]. Fourth, it is difficult to deliver the antioxidants such as N-acetylcysteine into cells and reach mitochondria in the CNS by crossing the blood brain barrier. The ideal antioxidant not only wipe off harmful ROS, but also can be used in clinical practice.

Hydrogen is potentially such an ideal antioxidant. Firstly, the reactivity of H_2_ is so mild that H_2_ only reacts with the strongest oxidants. Hydrogen does not disturb the metabolic oxidation-reduction reactions or disrupt ROS involved in cell signaling and the defense system against harmful stimulation. Hydrogen also has no effect on physiology, temperature, blood pressure, pH, or pO_2_[25]. Second, Hydrogen can diffuse rapidly into tissues and cells, and readily reach subcellular compartments[26]. Hydrogen can immediately scavenge •OH, when it is produced. Third, Hydrogen has not been reported to be toxic at effective dosages and even at high concentration[27]. The excess H_2_ would be expired via the lungs when too much is taken in. Moreover, clinical studies have shown that H_2_ can treat many diseases associated with oxidative stress by scavenging •OH in patients[28], not only in cells[6] and explant cultures[19] as well as animals[20]. Studies showed that Hydrogen-rich water improves lipid and glucose metabolism in patients with type 2 diabetes or impaired glucose tolerance[29]. Hydrogen can be delivered in several formulations, such as inhaling H_2_, drinking H_2_-dissolved water (H_2_-water), injecting H_2_-dissolved saline (H_2_-saline), taking an H_2_ bath, or dropping H_2_-saline into the eyes[30], and taking in medicine that promoted the production of H_2_[28]. In the current study, our data is consistent with these previous studies. Therefore, Hydrogen is potentially an ideal antioxidant in clinics. Oxygen is oxidative and hydrogenHydrogen is reductive. Hydrogen is prone to react with oxygen, resulting in burning and explosion. However, Hydrogen is flammable only at temperatures higher than 527°C, and explodes upon a rapid chain reaction with O_2_ only in the explosive range of H_2_ concentration (4–75%, vol/vol). In our study, we used 2% (v/v%) H_2_, well lower the concentration for burning or explosion with oxygen. The safety of using mixed 2% H_2_ and O_2_ has been proved through our work and other studies[31]. Therefore, Hydrogen can be safely applied to medical applications, although the use of H_2_ must be monitored and maintained with an approved and commercially available tool in case of danger.

## 6. Conclusion

We have found that Hydrogen gas prevents oxygen toxicity, alleviates PC12 cellular injury, and improves the survival rate of PC12 cells probably through selectively neutralizing •OH generated from hyperbaric oxygen and decreasing the levels of damaged biomacromolecule. Our findings supported that H_2_ could be potentially used as an antioxidant during HBO therapy.
